# Saikosaponin a Enhances Transient Inactivating Potassium Current in Rat Hippocampal CA1 Neurons

**DOI:** 10.1155/2013/413092

**Published:** 2013-02-21

**Authors:** Wei Xie, Yun Hong Yu, Yong Ping Du, Yun Yan Zhao, Chang Zheng Li, Lin Yu, Jian Hong Duan, Jun Ling Xing

**Affiliations:** ^1^Department of Traditional Chinese Medicine, Nanfang Hospital, Southern Medical University, Guangzhou, Guangdong 510515, China; ^2^School of Traditional Chinese Medicine, Southern Medical University, Guangzhou, Guangdong 510515, China; ^3^Department of Traditional Chinese Medicine, Xijing Hospital, Fourth Military Medical University, Xi'an, Shanxi 710032, China; ^4^Intensive Care Unit, Guang Zhou Municipal Hospital of Chinese Medicine, Guangzhou, Guangdong 510130, China; ^5^Department of Traditional Chinese Medicine, Guang Zhou Brain Hospital, Guangzhou, Guangdong 510170, China; ^6^Institute of Neuroscience, Fourth Military Medical University, Xi'an, Shanxi 710032, China

## Abstract

Saikosaponin a (SSa), a main constituent of the Chinese herb *Bupleurum chinense* DC., has been demonstrated to have antiepileptic activity. Recent studies have shown that SSa could inhibit NMDA receptor current and persistent sodium current. However, the effects of SSa on potassium (K^+^) currents remain unclear. In this study, we tested the effect of SSa on 4AP-induced epileptiform discharges and K^+^ currents in CA1 neurons of rat hippocampal slices. We found that SSa significantly inhibited epileptiform discharges frequency and duration in hippocampal CA1 neurons in the 4AP seizure model in a dose-dependent manner with an *IC*_50_ of 0.7 **μ**M. SSa effectively increased the amplitude of *I*_Total_ and *I*_*A*_, significantly negative-shifted the activation curve, and positive-shifted steady-state curve of *I*_*A*_. However, SSa induced no significant changes in the amplitude and activation curve of *I*_*K*_. In addition, SSa significantly increased the amplitude of 4AP-sensitive K^+^ current, while there was no significant change in the amplitude of TEA-sensitive K^+^ current. Together, our data indicate that SSa inhibits epileptiform discharges induced by 4AP in a dose-dependent manner and that SSa exerts selectively enhancing effects on *I*_*A*_. These increases in *I*_*A*_ may contribute to the anticonvulsant mechanisms of SSa.

## 1. Introduction 

**Figure 1:**
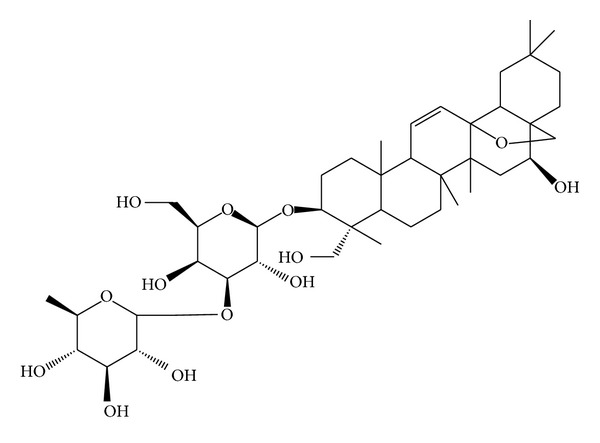
Chemical structure of Saikosaponin a (SSa).

Saikosaponin a (SSa) is the primary active constituent derived from *Bupleurum chinense *DC. (Umbelliferae) [1]. The molecular formula of SSa is C_42_H_68_O_13_ with a molecular weight of 780.98. The molecular structure of SSa is shown in Figure 1. In clinical and laboratory studies, it has been reported that SSa has a variety of pharmacological benefits, including anti-inflammatory, immunomodulatory, and antibacterial activities [2]. In recent years, we have found that SSa demonstrates antiepileptic activities in a variety of in vivo seizure models including the pentylenetetrazole kindling model, the maximal-electroshock-(MES-) induced seizure model and the Li-Pilocarpine-induced refractory epilepticus [3–5]. Our recent studies have shown that SSa inhibits spontaneous recurrent epileptiform discharges (SREDs) and continuous tonic high-frequency epileptiform bursts (SE) induced by applying Mg^2+^-free solution in hippocampal neuronal cultured models of acquired epilepsy and status epilepticus [6]. In addition, SSa could inhibit NMDA receptor current and persistent sodium current in cultured hippocampal neurons [6]. However, we have also found that the half-maximum effective dose of SSa required to inhibit SE is larger than that required to inhibit SREDs in the low-Mg^2+^ seizure model in vitro [6]. This evidence suggests that SSa anticonvulsant activities may result from other mechanisms, in addition to the inhibitions of NMDA receptor current and persistent sodium current.

Under physiological conditions, voltage-gated potassium (K^+^) currents are key regulators of neuronal excitability [7], such as resting membrane potential, action potential waveform, and firing frequency [8, 9]. Alterations in their electrical function are related to hyperexcitability and epilepsy [10, 11]. Several animal models of epilepsy have been developed by using K^+^ channel blockers, such as 4-aminopyridine (4AP) [12], dendrotoxin I [13], tityustoxin-K, and pandinustoxin-K [14]. In addition, mice with induced deletions of various K^+^ current genes exhibit seizures [15, 16]. K^+^ currents were decreased in hippocampal CA1 pyramidal neuron dendrites in several animal models of seizure [17, 18]. Drugs that enhance K^+^ currents activity possess antiepileptic action [19, 20]. Thus, K^+^ currents may play an active role in controlling epileptic synchronization [21, 22]. 

In hippocampal CA1 neurons, the total voltage-gated outward K^+^ current (*I*_Total_) consists of two main types of voltage-gated K^+^ currents, a transient inactivating K^+^ current (*I*_*A*_) and a delayed rectifier K^+^ current (*I*_*K*_) [8, 9]. As *I*_*A*_ is transient, repolarization is mainly related to *I*_*K*_, while *I*_*A*_ plays an important role in repetitive firing and back propagation of action potential into dendrites [23]. *I*_*A*_ and *I*_*K*_ play major roles in regulating the excitability of neurons [7]. Changes in their kinetic properties can alter neuronal excitability and electrical function [24].

In the present study, we used whole-cell patch-clamp recordings to evaluate the anticonvulsant effects of SSa on 4AP-induced epileptiform discharges and to investigate the dynamic and pharmacological modulation effects of SSa on K^+^ currents in CA1 neurons of rat hippocampal slice. Using whole-cell recording techniques, we found that SSa inhibited epileptiform discharges induced by 4AP in a dose-dependent manner and that SSa exerts selectively increasing effects on *I*_*A*_ but not *I*_*K*_ in hippocampal CA1 neurons. The increasing effects on *I*_*A*_ may contribute to the anticonvulsant mechanisms of SSa.

## 2. Materials and Methods

### 2.1. Materials

Saikosaponin a (purity > 98%, obtained from Shanghai Institute of Pharmaceutical Industry, Shanghai, China) was dissolved in DMSO at a concentration of 100 mM as a stock solution and stored at −20°C. Working solutions were prepared before each experiment, and the final DMSO concentration did not exceed 0.1% throughout the study. Recordings were performed in the presence and absence of SSa or the vehicle. Other reagents were purchased from Sigma (St. Louis, MO, USA). 

### 2.2. Slice Preparation

Sprague-Dawley rats (25–50 days old) were provided by the Animal Center of the Fourth Military Medical University. The animals were housed and handled in strict accordance with the guidelines of the institutional and national Committees of Animal Use and Protection. The protocol was approved by the Committee on the Ethics of Animal Experiments of the Fourth Military Medical University. Hippocampal slices were cut in this study as previously described [25], with some modifications. Rats were anesthetized with isoflurane and decapitated. The brains were rapidly removed and placed in ice-cold Ringer's solution that contained (in mM) 124 NaCl, 2.5 KCl, 1.25 NaH_2_PO_4_, 2 MgSO_4_, 25 NaHCO_3_, 0.5 CaCl_2_, and 10 glucose and was gassed with 95% O_2_/5% CO_2_ mixture to attain a pH of 7.2–7.4. Transverse brain slices (300–350 *μ*m in thickness) that included the entire hippocampus were cut with a Vibratome (VT1000S, Leica, Germany) and left undisturbed in an incubation chamber for 1 h for stabilization at room temperature (21–24°C) in artificial cerebrospinal fluid (ACSF) that contained (in mM) 124 NaCl, 2.5 KCl, 1.25 NaH_2_PO_4_, 2 MgSO_4_, 25 NaHCO_3_, 2 CaCl_2_, and 10 glucose and was gassed with 95% O_2_/5% CO_2_.

### 2.3. Electrophysiological Recordings

Hippocampal CA1 pyramidal neurons were visualized with an Olympus BX51WI upright microscope (Olympus Optical Co., Center Valley, PA, USA) equipped with a 40X water-immersion lens and optics for differential interference contrast and infrared (DIC-IR). Recorded cells were identified as CA1 hippocampal pyramidal neurons according to their placement in the pyramidal layer and their electrophysiological properties [26]. Patch electrodes with a resistance of 5–8 MΩ in the bath were pulled on a microelectrode puller (P97, Sutter Instruments, USA). The epileptiform activity was recorded in whole-cell current-clamp mode and potassium currents were collected in whole-cell voltage-clamp mode. Whole-cell current- and voltage-clamp recordings were carried out using an Axopatch 700B amplifier (Axon Instruments, Foster City, CA). The data were transferred to a PC using a Digidata 1322A (Axon Instruments) interface and acquired using pCLAMP 9 (Axon Instruments) software. All junction potentials were corrected online by adjusting the pipette offset using the 700B Commander software (Molecular Devices). 

For whole-cell current-clamp recordings, slices were placed in an immersion recording chamber and superfused (1.5 mL/min) with gassed ACSF at 32-33°C. The recording pipette solution contained (in mM) 140 K gluconate, 5 NaCl, 1 CaCl_2_, 2 MgCl_2_, 11 EGTA, 10 HEPES, 2 Mg-ATP, and 0.3 Na-GTP, pH 7.4 with KOH. To induce epileptiform events, slices were superfused with ACSF containing 100 mM 4AP for 20–40 min [27]. Recordings were obtained from hippocampal CA1 neurons. Once epileptiform discharges were present and stable for 20–40 min (at least three epileptiform discharges), SSa was superfused concurrent with the convulsant treatment for 20–40 min to evaluate the inhibition of epileptiform activities. Finally, a 20–40 min superfusion of ACSF contained 100 mM 4AP was performed as a washout for observations of any persistent effects. The data were digitized at 2 kHz.

To record K^+^ currents, 1 *μ*M tetrodotoxin (TTX) and 200 *μ*M cadmium chloride (CdCl_2_) were added into the ACSF to block Na^+^ and Ca^2+^ currents. After the establishment of a whole-cell voltage-clamp configuration, the cells were allowed to stabilize for 3–5 min before starting pulse protocols to record the currents as control. After the currents were stable, SSa (1 *μ*M) was added into the ACSF to examine its effects on the properties of voltage-gated K^+^ currents (*I*_Total_, *I*_*A*_ and *I*_*K*_) in rat hippocampal CA1 neurons. Modifications of 4AP-sensitive and TEA-sensitive K^+^ currents by SSa were also observed. Signals were low-pass filtered at 2 kHz and digitized at 10 kHz. Fast and slow capacitances were neutralized, and the series resistance was compensated (50–70%) and periodically monitored. Capacitance transients and leakage currents linear with voltage were subtracted using a P/4 voltage protocol. Recordings were made at room temperature (22–24°C). 

### 2.4. Statistical Analysis

All results were expressed as the mean ± SEM. Statistical analysis was performed using Statistical Product and Service Solutions (SPSS) software (paired *t*-test, unpaired *t*-test, or one-way ANOVA). Values of *P* < 0.05 were considered to be significant. The data were plotted using Origin 8.0.

Changes of epileptiform discharges frequency and duration were quantified. For concentration-response analysis, the percentage inhibition of epileptiform discharges frequency was determined at different concentrations of SSa. The concentration-response curve was obtained by fitting the experimental data with a logistic equation with variable slope (Hill coefficient): *y*/*y*_max⁡_ = {1 − [*D*/(*D*+*IC*_50_)]^*n*^}, where *y* is the response in the presence of drug, *y*_max⁡_ is the maximal response in the absence of drug, *D* is the drug concentration, *IC*_50_ is the concentration of drug producing a half-maximal inhibition of the response, and *n* is the Hill coefficient. Fit estimates were calculated using Marquardt nonlinear least squares algorithms. Steady-state activation and inactivation curves were fitted with the Boltzmann equation: *I*/*I*_max⁡_ = 1/[1 + exp⁡((*V*_*m*_−*V*_1/2_)^*k*^)], where *I*/*I*_max⁡_ is normalized current, *V*_*m*_ is the membrane potential of the preceding voltage step, *V*_1/2_ is the potential for half-maximal activation/inactivation, and *k* is the slope factor.

## 3. Results 

### 3.1. Effects of SSa on Epileptiform Discharges Induced by 4AP in Hippocampal CA1 Neurons in Hippocampus Slices

The in vitro hippocampal seizure model induced by 100 *μ*M 4AP was used to evaluate the anticonvulsant activities of SSa with whole-cell current-clamp recordings [27]. The anticonvulsant effects of SSa were observed at doses as low as 0.1 *μ*M and as high as 4 *μ*M. The percentage inhibition of epileptiform discharges frequency and duration was determined to evaluate the effectiveness of SSa. 

**Figure 2:**
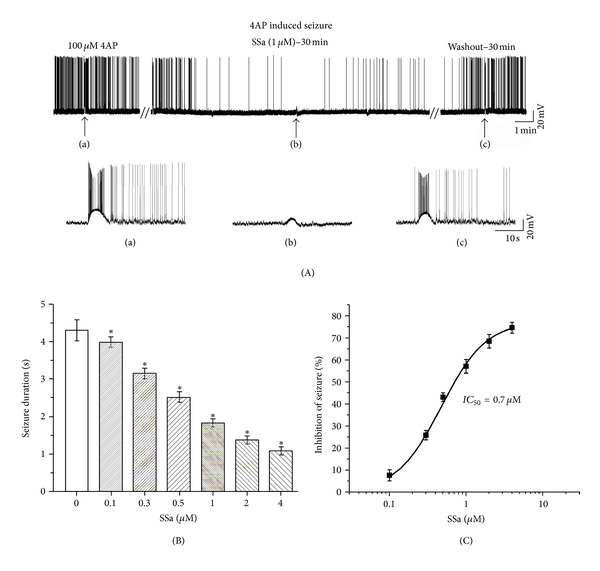
Effects of SSa on the epileptiform discharges induced by bath application of 100 *μ*M 4-aminopyridine (4AP) in hippocampal CA1 neurons in hippocampus slices. (A) A representative whole-cell current-clamp recording from a hippocampal CA1 neuron during superfusion of 100 *μ*M 4AP, after application of SSa (1 *μ*M), and during washout with ACSF containing 100 *μ*M 4AP. The trace has been truncated. Epileptiform discharges induced by 4AP are reduced in frequency and duration by bath application of SSa (1 *μ*M). Inserted segments (a–c) are expansions of the original traces at the arrows indicated and showing details of the inhibitory effect on epileptiform discharges activity. (B) Bar graph representing average duration of epileptiform discharges before and after application of increasing concentrations of SSa. (C) The log concentration-response curve for percentage inhibition of epileptiform discharges frequency after application of increasing concentrations of SSa. The *IC*_50_ was 0.70 *μ*M. Data are represented as mean ± SEM (*n* = 8). *P < 0.05 (*versus* control).

Superfusion of 100 *μ*M 4AP for 20–40 min caused a prominent increase in the duration of the action potential due to a slow repolarization phase (Figures 2(A)-(a)). The ictal discharges phase consisted of tonic and clonic components and was followed by interictal events. During the ictal tonic phase, the membrane depolarized to −20 mV ± 2.2 mV (*n* = 8) and gradually recovered within 5 s of the ictal clonic phase to the control value of −65.3 ± 3.1 mV (*n* = 8). Therefore, epileptiform discharges can be generated in the intact preparation by 4AP with all the phases that are observed in vivo. After coapplication of SSa (1 *μ*M) with 100 *μ*M 4AP for 20–40 min, the epileptiform discharges duration was significantly decreased from 4.3 ± 0.28 s (*n* = 8) in 4AP solution to 1.8 ± 0.1 s (*n* = 8; paired *t*-test; *P* < 0.01; Figures 2(A)-(b), (c)). The epileptiform discharges frequency was significantly decreased by 57.3 ± 3.1% (*n* = 8) by SSa at dose of 1 *μ*M (Figure 2(C)). Washing out the drug with ACSF containing 100 *μ*M 4AP >30 min, the epileptiform discharges duration and frequency returned to the values observed before SSa. The log concentration-response curve of percentage inhibition of epileptiform discharges frequency showed that SSa inhibited epileptiform activities in a concentration-dependent manner and that the *IC*_50_ was 0.70 *μ*M (Figure 2(C)).

### 3.2. Effects of SSa on *I*_Total_, *I*_*K*_, and *I*_*A*_

**Figure 3:**
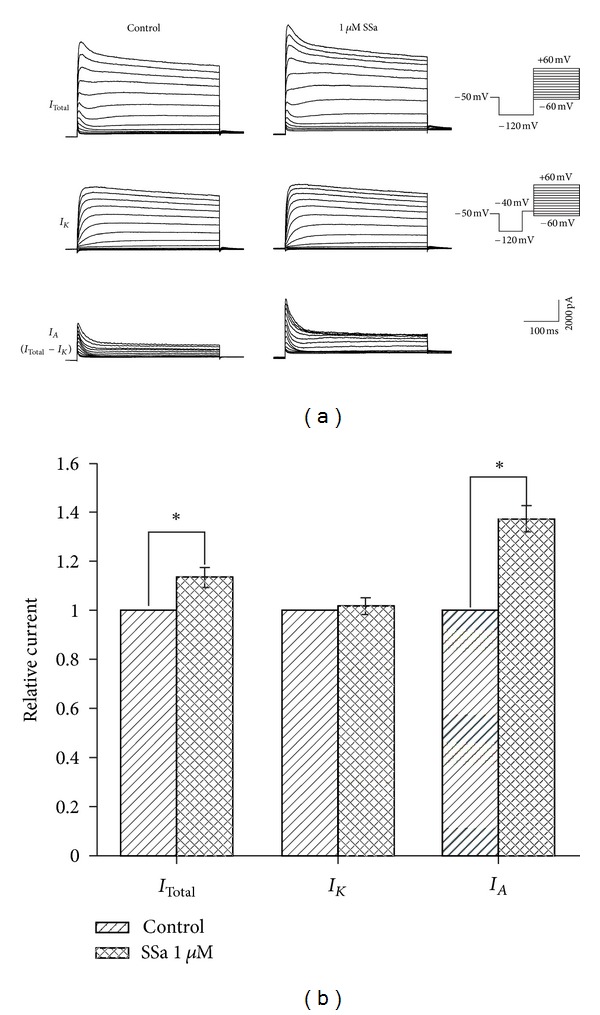
Effects of SSa on *I*_Total_, *I*_*K*_, and *I*_*A*_. (a) Typical traces of three voltage-gated outward K^+^ currents, namely *I*_Total_, *I*_*K*_, and *I*_*A*_, before (control, left panel) and after (middle panel) application of 1 *μ*M SSa. The pulse protocols are shown on the right. *I*_*A*_ was determined by point-by-point subtracting *I*_*K*_ from *I*_Total_. (b) Bar graph showing the effects of SSa (1 *μ*M) on *I*_Total_, *I*_*K*_, and *I*_*A*_ by normalizing the peak currents to the control under each condition. Data are represented as mean ± SEM (*n* = 8). *P < 0.05 (*versus* control).

**Figure 4:**
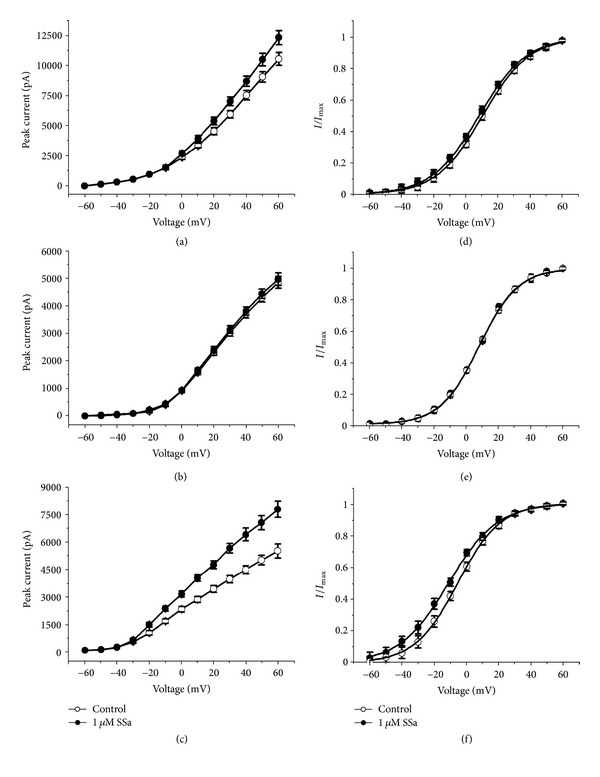
Effects of SSa on *I-V* relationship and the activation kinetics of *I*_Total_, *I*_*K*_, and *I*_*A*_. (a, b, c) *I-V* relationships for *I*_Total_, *I*_*K*_, and *I*_*A*_, respectively, before and after application of 1 *μ*M SSa. (d, e, f) Steady-state activation curves for *I*_Total_, *I*_*K*_, and *I*_*A*_, respectively, determined by the Boltzmann equation under control conditions and after application of 1 *μ*M SSa. Data are represented as mean ± SEM (*n* = 8). *P < 0.05 (*versus* control).

The voltage protocols in Figures 3 and 4 were used for kinetic separation of the outward K^+^ currents. To elicit *I*_Total_, the holding potential was −50 mV and a 300 ms hyperpolarizing prepulse to −120 mV was followed by a series of 400 ms steps from −60 to +60 mV in 10 mV increments, delivered every 10 s (Figure 3(a), top of right panel). To elicit *I*_*K*_, a similar protocol was used, but a 100 ms interval at −40 mV was inserted after the prepulse (Figure 3(a), middle of right panel). *I*_*A*_ was calculated by point-by-point subtracting *I*_*K*_ from *I*_Total_ [28]. Figure 2(A) shows typical traces of three outward voltage-gated K^+^ currents (*I*_Total_, *I*_*K*_, and *I*_*A*_) before (control, left panels) and after (middle panels) administration of 1 *μ*M SSa. Normalizing the peak current, SSa produced only a small increase in the amplitude of *I*_Total_, by 13.6 ± 0.2% (*n* = 8; paired *t*-test; *P* < 0.05; Figure 3(b)) and did not induce a significant change in the amplitude of *I*_*K*_ (*n* = 8; paired *t*-test; *P* > 0.05). SSa at 1 *μ*M caused an obvious increase in the amplitude of *I*_*A*_ after subtracting *I*_*K*_ from *I*_Total_, by 37.7 ± 0.5% (*n* = 8; paired *t*-test; *P* < 0.05; Figure 3(b)). 

### 3.3. Effects of SSa on *I-V* Relationship and the Activation Kinetics of *I*_Total_, *I*_*K*_, and *I*_*A*_

The *I-V* relationships for *I*_Total_,  *I*_*K*_, and *I*_*A*_ under control condition and after application of 1 *μ*M SSa are shown in Figures 4(a), 4(b), and 4(c), and the steady-state activation curves for *I*_Total_, *I*_*K*_, and *I*_*A*_ are shown in Figures 4(d), 4(e), and 4(f). The increasing effects of SSa on the amplitude of *I*_Total_ and *I*_*A*_ were voltage dependent. As the membrane potential was stepped to more depolarizing values, the amplitude-increasing effects of SSa increased nominally (Figures 4(a), 4(c)), while there was no significant change in *I*_*K*_ (Figure 4(b)). There was no significant change in voltage for half-maximal activation (*V*_1/2_) of *I*_Total_ between control and SSa-treated preparations (32.4 ± 2.6 versus 30.0 ± 3.1 mV, respectively; *n* = 8; paired *t*-test; *P* > 0.05), nor did the slope factor (*k*) change (33.5 ± 2.5 versus 32.1 ± 3.2, respectively; *n* = 8; paired *t*-test; *P* > 0.05). Similarly, no significant change was observed in *V*_1/2_ for *I*_*K*_ in control and SSa-treated preparations (28.2 ± 6.2 versus 28.5 ± 7.9 mV, respectively; *n* = 8; paired *t*-test; *P* > 0.05) as well as *k* (30.5 ± 4.1 versus 31.0 ± 3.7, respectively; *n* = 8; paired *t*-test; *P* > 0.05). SSa caused a significant change in *V*_1/2_ for *I*_*A*_ (28.7 ± 2.1 in control versus 22.1 ± 1.3 mV in SSa; *n* = 8; paired *t*-test; *P* < 0.05) with *k* (21.9 ± 3.2 versus 24.2 ± 2.6, respectively; *n* = 8; paired *t*-test; *P* > 0.05). The steady-state activation curves showed that SSa significantly negative-shifted the voltage dependence of the activation of *I*_*A*_ with no change in slope factor (Figure 4(f)).

### 3.4. Effects of SSa on the Steady-State Inactivation Properties of *I*_*A*_

**Figure 5:**
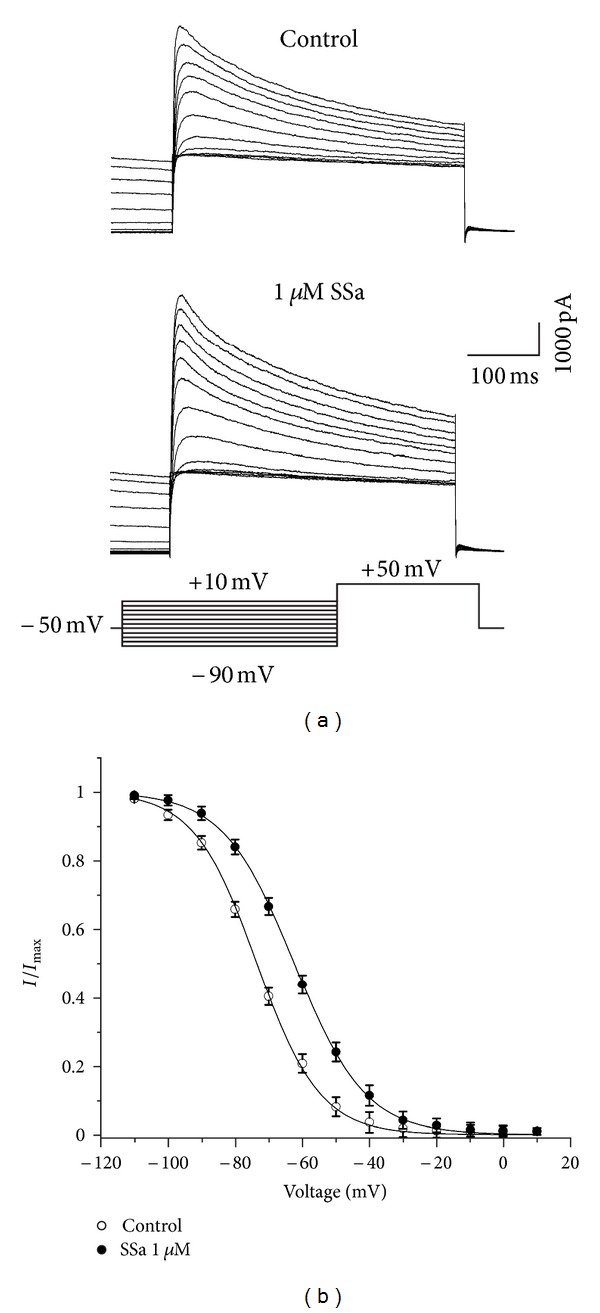
Effects of SSa on the steady-state inactivation properties of *I*_*A*_. (a) Typical traces of *I*_*A*_, under control condition (top panel) and after application of 1 *μ*M SSa (middle panel). Currents were elicited with a series of 120 ms prepulses at different hyperpolarizing potentials between −90 mV and +10 mV in 10 mV increments, followed by an 80 ms depolarizing pulse to +50 mV, delivered every 10 s (bottom panel). Currents at the end of the depolarizing pulse represented *I*_*A*_. (b) The steady-state inactivation curves for *I*_*A*_ in the absence and presence of 1 *μ*M SSa. The peak amplitudes of *I*_*A*_ were normalized and plotted against the prepulse potentials and the data were fitted with the Boltzmann function. Data are represented as mean ± SEM (*n* = 8). *P < 0.05 (*versus* control).

Steady-state inactivation was studied based on the double-pulse protocols as follow. Neurons were held at −50 mV and currents were elicited with an 80 ms test pulse to +50 mV preceded by 120 ms prepulses to potentials between −90 mV and +10 mV (Figure 5(a), bottom panel). The peak amplitudes of *I*_*A*_ were normalized and plotted versus the prepulse potentials. The curves were fitted with the Boltzmann equation, as described in Materials and Methods: *I*/*I*_max⁡_ = 1/[1 + exp⁡((*V*_*m*_−*V*_1/2_)^*k*^)]. Application of 1 *μ*M SSa caused a significant depolarizing shift of the voltage-dependent steady-state inactivation of *I*_*A*_ (Figure 5(b)). *V*_1/2_ of *I*_*A*_ changed from −73.4 ± 1.36 mV in control to −62.81 ± 0.94 mV in SSa (*n* = 8; paired *t*-test; *P* < 0.05), with the slope factor, *k*, values of 9.4 ± 0.4 and 12.6 ± 0.8, respectively (*n* = 8; paired *t*-test; *P* > 0.05).

### 3.5. Effects of SSa on 4AP-Sensitive and TEA-Sensitive K^+^ Current

**Figure 6:**
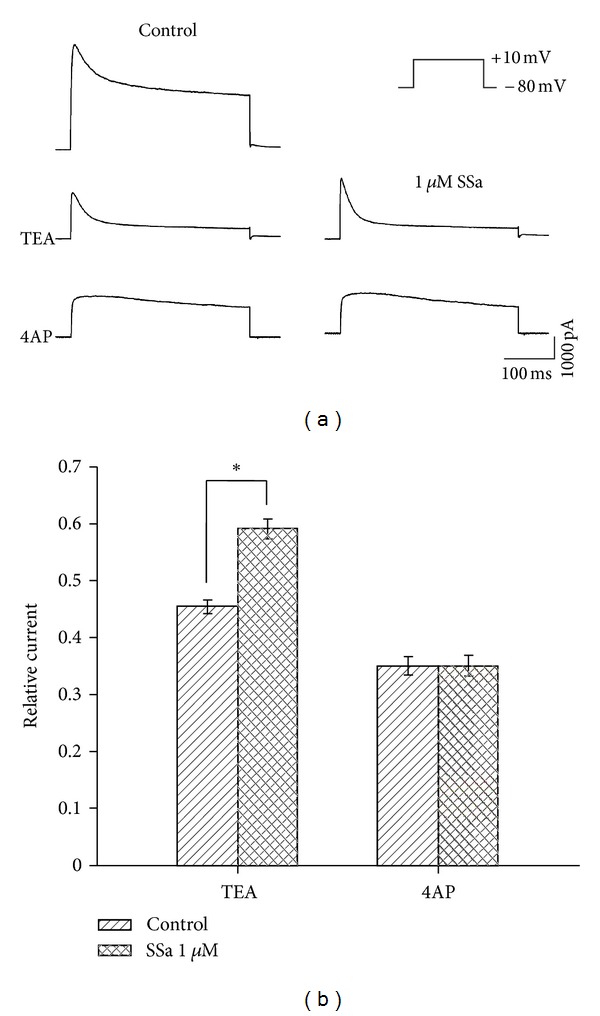
Effects of SSa on 4AP-sensitive and TEA-sensitive K^+^ currents. (a) Representative recordings from a hippocampal CA1 neuron before (left panel) and after applying 1 *μ*M SSa (right panel) during application of 20 mM TEA (middle panel) or 4 mM 4AP (bottom panel). Outward K^+^ currents were induced by a depolarizing command from −80 mV to +10 mV (right of top panel). (b) Bar graph showing the effects of SSa (1 *μ*M) on 4AP-sensitive and TEA-sensitive K^+^ currents by normalizing the peak currents with control under each condition. Data are represented as mean ± SEM (*n* = 8). *P < 0.05 (*versus* control).

*I*_*A*_ is sensitive to high concentration of 4AP, whereas *I*_*K*_ is blocked by TEA [29]. Hence, we further established whether SSa was capable of modulating one or both of these two pharmacologically distinct, outward K^+^ currents. Outward K^+^ currents were generated by a depolarizing pulse +10 mV delivered from a holding potential of −80 mV (Figure 6(a), right of top panel). 4AP-sensitive (Figure 6(a), middle panel) and TEA-sensitive K^+^ current (Figure 6(a), bottom panel) were obtained by application of 20 mM TEA or 4 mM 4AP, respectively. SSa enhanced a transient, 4AP-sensitive outward K^+^ current in the presence of 20 mM TEA by 13.6 ± 0.2% (normalized with control; *n* = 8; paired-*t* test; *P* < 0.05; Figure 6(b)), while it did not induce a significant modification in the amplitude of a late, TEA-sensitive K^+^ current after application of 4 mM 4AP (Figure 6(b)).

## 4. Discussion 

The present study indicated, for the first time, that SSa inhibited epileptiform events induced by 100 *μ*M 4AP in rat CA1 neurons in hippocampus slices in a dose-dependent manner. In the 4AP seizure model, SSa showed long-lasting effects by decreasing the seizure amplitude for >30 min with washout, similar to other anticonvulsants such as carbamazepine [30]. In recent studies, we have found that SSa inhibited epileptiform discharges evoked by low-Mg^2+^ solution in hippocampal neuronal cultured models of acquired epilepsy and status epilepticus [6]. These results demonstrate that SSa has anticonvulsant properties in both the 4AP seizure model and the low-Mg^2+^ seizure model. As is known, some anticonvulsants appear to be more effective in some models of seizures than in others. For instance, when examining the efficacy on refractory seizures induced by low Mg^2+^ in the immature corticohippocampal formation in vitro, some drugs such as valproate suppress epileptiform activity, whereas ethosuximide, gabapentin, phenytoin, and topiramate are ineffective [31]. Valproate, and not phenytoin or carbamazepine, abolishes epileptiform activity induced by 4AP in entorhinal cortex-hippocampal slices [32]. These results may imply SSa as a novel effective treatment for epilepsy. 

The inhibitions of NMDA receptor current and persistent sodium current in cultured hippocampal neurons may be some anticonvulsant mechanisms of SSa [6]. While the results that the percentage inhibition of epileptiform discharges frequency at the same dose of SSa in 4AP-induced seizure were different from that in low-Mg^2+^-induced seizure, which led us to consider the possibility that the different sensitivities of these two types of epileptiform discharges to SSa are caused by an additional mechanism of action. As 4AP acts as a convulsant, resulting from the blockade of K^+^channels [12], the modulation of K^+^ currents by SSa was attractive.

From further studies in rat hippocampal CA1 neurons, we found that SSa effectively increased the amplitude of *I*_Total_ and *I*_*A*_. SSa significantly shifted the activation curves of *I*_*A*_ to negative potentials. In addition, SSa significantly positive-shifted the steady-state inactivation of *I*_*A*_. However, there were no significant effects of SSa on the amplitude and activation curves of *I*_*K*_, which might account for the result that the increasing potency of SSa was much stronger with regards to *I*_*A*_ than *I*_*K*_. These results suggest that SSa has a selective modification effect on *I*_*A*_. 

*I*_*A*_, prominent in hippocampal neurons, shows fast activation and relatively fast inactivation [8, 33]. It belongs to the threshold currents activated near the spiking threshold [34], and in this respect, it delays the action potential and controls the interspike interval during repetitive firing [29, 35]. *I*_*A*_ regulates repetitive firing, which is obviously related to abnormal discharge [36, 37]. The reduction of *I*_*A*_ will induce neuronal hyperexcitability and finally result in epilepsy [37, 38]. 4AP, dendrotoxin I, tityustoxin-K, or pandinustoxin-K are known to readily induce epileptiform discharges resulting from blockade of K^+^ channels [10, 12, 14]. In addition, recent evidence indicates that some epileptic syndromes occurring in both animals and humans are caused by mutations in K^+^ channel genes [15, 16]. In hippocampal tissue after Li-Pilocarpine-induced seizures, CA1 pyramidal neuron dendrites showed decreased availability of the *I*_*A*_ due to transcriptional and posttranslational processes [39, 40]. *I*_*A*_ has the property to selectively suppress rapid, large, and synchronized EPSPs inputs [41, 42]. It may thus limit the intensity of firing and reduce the duration of epileptiform discharges, which may be an important mechanism for the control of seizures. Indeed, several anticonvulsants have been shown to enhance *I*_*A*_ including lamotrigine [43, 44], valproate [45], and carbamazepine [46]. Hence, the effects of SSa on *I*_*A*_ reported here may be relevant for its anticonvulsant action. 

*I*_*A*_ can be completely blocked by high concentrations of 4AP, while *I*_*K*_ is highly sensitive to TEA [29, 47]. We further evaluated the modulatory effect of SSa on 4AP-sensitive K^+^ current and TEA-sensitive K^+^ current, isolated pharmacologically. We found that SSa significantly increased the outward K^+^ current during application of 20 mM TEA, while there was no obvious change in the outward K^+^ current during application of 4 mM 4AP. This result suggests that SSa possesses a high affinity to 4AP-sensitive K^+^ current, which maybe consistent with the above finding that SSa selectively increased *I*_*A*_. 

In conclusion, our results demonstrate that SSa exerts anticonvulsant properties by inhibiting 4AP-induced epileptiform discharges in CA1 neurons in rat hippocampus slices. Increase of *I*_*A*_, but not *I*_*K*_, by SSa may represent a mechanism by which SSa exerts anticonvulsant properties. However, several issues remain to be investigated in the future. First is the lack of functional data providing a direct link between K^+^ channel increase and direct indicators of the anticonvulsant properties of SSa. Second, studies on heterologously expressed genes encoding the ion channels responsible for *I*_*A*_ and *I*_*K*_ could be used to distinguish the effects of SSa. Third, studies evaluating the effects of SSa on other types K^+^current, such as M-type K^+^ current, would be performed to investigate other anticonvulsant mechanisms of SSa. 

## Abbreviations

SSa: Saikosaponin a
4AP: 4-aminopyridine
SREDs: Spontaneous recurrent epileptiform discharges
SE: Continuous tonic high-frequency epileptiform bursts
*I*_Total_: Total voltage-gated outward K^+^ current
*I*_*A*_: Transient inactivating K^+^ current
*I*_*K*_: Delayed rectifier K^+^ current
ACSF: Artificial cerebrospinal fluid.
